# MEMS Shielded Capacitive Pressure and Force Sensors with Excellent Thermal Stability and High Operating Temperature

**DOI:** 10.3390/s23094248

**Published:** 2023-04-25

**Authors:** Muhannad Ghanam, Frank Goldschmidtboeing, Thomas Bilger, Andreas Bucherer, Peter Woias

**Affiliations:** Laboratory for Design of Microsystems, IMTEK—University of Freiburg, 79106 Freiburg im Breisgau, Germany; muhannad.ghanam@imtek.de (M.G.); frank.goldschmidtboeing@imtek.uni-freiburg.de (F.G.); thomas.bilger@web.de (T.B.); andreas.bucherer@imtek.uni-freiburg.de (A.B.)

**Keywords:** capacitive pressure sensors, capacitive force sensors, high-temperature applications, temperature drift

## Abstract

In this paper, we present an innovative manufacturing process for the production of capacitive pressure and force sensors with excellent thermal stability for high-temperature applications. The sensors, which are manufactured from a stack of two silicon chips mounted via with gold–silicon (Au-Si) or aluminum–silicon (Al-Si) eutectic bonding, are shielded, miniaturized, and allow an operating temperature of up to 500 °C. Compared to conventional methods, the greatest benefit of the manufacturing process is that different sensor dimensions can be produced in the same batch for a wide measuring range, from mN to kN. The characterization of the realized sensors shows a high linearity and a low temperature drift of 99.992% FS and −0.001% FS/K at 350 °C, as well as a nonlinearity of 0.035% FS and a temperature drift of −0.0027% FS/K at 500 °C.

## 1. Introduction

The continuous monitoring and evaluation of process parameters in industry is of great importance in order to control technical systems and to detect and avoid errors and possible failures at an early stage. Pressure and force sensors play an important role for this task. Both their development and fabrication fall into a classical field of microsystems technology with mature products and high sales. It may therefore come as a surprise at first glance that there is still a need for further research in this “mature” area of micro-electro-mechanical systems (MEMS). However, many applications, such as environmental monitoring industrial process control, aerospace, petrochemical, and combustion process monitoring in power generation plants [1,2,3], operate in harsh environments such as high temperatures. Conventional sensor technology often fails at such temperatures and cannot withstand all the harsh requirements of such applications, such as higher operating temperatures, higher thermal and mechanical stability, and higher sensitivity [4,5]. To this end, Ref. [6] prioritizes the following scientific and industrial requirements and development goals:

- Increase of the usable pressure and temperature range,
- Increase of the long-term stability of the sensor signal,
- Improvement of the media resistance,
- Reduction of the temperature dependence of the sensor signals.

In addition, cost reduction is, of course, always desirable. The most known technologies for realization of pressure and force sensors are optical, piezoelectric, piezoresistive, and capacitive methods [7], which provide a broad spectrum of solution concepts but will not address all requirements given above. Therefore, despite the advanced development in the area of pressure and force sensors, industry is constantly looking for new MEMS solutions that can meet these requirements. In the following, we discuss the available technologies, and their ability to fulfill the requirements given above.

### 1.1. Optical Strain Gauges

Optical strain gauges or fiber optic sensors (FOSs) can be used to measure various physical quantities, for example, acceleration, strain, humidity, shape, and temperature [8,9,10,11]. Unlike conventional electrical sensors, FOSs do not need to be supplied with electrical power. This makes them completely passive and insensitive to electromagnetic interference (EMI) [11,12]. For these reasons, FOSs have become increasingly important in recent years. The measurement principle is based on the change of the refractive index of a fiber Bragg grating (FBG). If a force or pressure acts on the FOS, this leads to an expansion or compression of the FBG and thus to a change in the refractive index. However, since optical fibers are inherently very temperature-sensitive, measuring slowly varying strain fields with temperature changes is a major challenge [9]. Typically, a temperature change of 1 K has the same effect as a strain change of 10 με. Therefore, when using an FOS, measurements without temperature compensation are not conceivable. In 2022, Tian et al. presented [8] the results of an FOS based on two cascaded thermally regenerated fiber Bragg gratings (RFBGs) for the simultaneous measurement of temperature and strain in a high-temperature environment. The RFBGs are mounted on two fused silica capillary tubes, where the RFBG2 is exclusively temperature-sensitive, while the RFBG1 remains sensitive to both strain and temperature. The measurements show a thermal sensitivity of about 15.7 pm/°C in the temperature range of 100–1000 °C, and for the RFBG1, a strain sensitivity up to about 5.46 pm/με in the measurement range of 0 με to 120 με at 600 °C. The thermal sensitivity is many times higher than the pressure sensitivity, which makes a reasonable measurement without compensation impossible.

### 1.2. Piezoelectric Sensors

Piezoelectric sensors convert mechanical stress into an analog voltage [13,14,15,16,17]. Forces or pressures deform the piezoelectric crystal structure and, thus, change the internal dipole moment of the piezoelectric material. This will induce a change in the electrical surface potential, proportional to the acting force. Thus, piezoelectric sensors are self-powered; i.e., they have very low energy requirements. The piezoelectric effect occurs in all ferroelectric materials and non-centrosymmetric crystal structures, such as quartz, aluminum nitride (AlN), lithium niobate (LN), lead zirconate titanate (PZT), etc. [16,18]. Piezoelectric sensors are known for their high sensitivity, accuracy, and robustness, and they are suitable for wide measurement ranges (typically from 0.7 kPa to 70 MPa) [7]. Natural crystals typically offer the widest temperature range and the lowest (or no) pyroelectric effect [19]. Artificially produced polarized polycrystalline ferroelectric ceramics such as bismuth titanate (BiTi) have a signal yield that is three-to-four times higher than natural crystals and can operate at temperatures up to 510 °C. In [19], an accelerometer for vibration and shock measurements in gas turbines was presented. The sensing element is a UHT-12^TM^ (ultra-high temperature 1200 °F/650 °C) piezoelectric crystal. In the data sheet of the EX611A20 model with a UHT-12^TM^ element [20], the sensor specifies a sensitivity of 1.02 pC/(m/s^2^) and a nonlinearity of ≤1%. The typical thermal sensitivity is ≤5% at 650 °C. However, the very low signal yield of a piezoelectric crystal, typically between 1 and 700 pC/N [21], requires a complex electrical interface. In order to minimize the loss of the output signal due to the high impedance, the evaluation circuit must be placed as close as possible to the sensor, which complicates sensor integration, especially in high-temperature applications [7]. Another important disadvantage of piezoelectric sensors is that they do not allow for static measurements.

### 1.3. Piezoresistive Sensors

Piezoresistive sensors are the oldest and most commonly used type of force and pressure sensors. Typically, the resistive elements are diffused, implanted, or deposited directly onto a stress-sensitive membrane [7,22]. As a result, the output signal is proportional to the induced stress in the membrane. They are characterized by high sensitivity, high linearity, and simple signal processing [23]. However, the main disadvantages of piezoresistive sensors are the relatively high power consumption and the temperature dependence of the sensor output, which must be compensated by electronic means. Since piezoresistive sensors are stress-sensitive, the output signal can be affected by mechanical or thermally induced stresses due to a mismatch between the sensor and its housing material. Moreover, they are generally limited by the leakage currents at the insulating pn-junctions, which increase with the increasing temperature, so that the maximum operating temperature of a silicon pressure sensor or force sensor is usually around 125 °C [24]. With the use of silicon-on-insulator (SOI) and silicon carbide (SiC), pressure sensors with a temperature range up to about 350 °C can be realized, but with much higher costs and more complex fabrication processes [25]. Peng et al. demonstrated in [26] an elaborate process for the fabrication of piezoresistive pressure sensor by using SOI. The p-type piezoresistive resistors were patterned on the <100> device wafer, while on the <111> handle wafer, the sensing diaphragm and a pressure reference chamber were fabricated. The measurements show an output signal of 180 mV/1500 kPa/10 VDC and a thermal hysteresis of 0.082% FS at the maximum temperature of 200 °C. In [27], Fraga et al. compared two in-house developed piezoresistive bending sensors fabricated from non-stoichiometric amorphous silicon carbide (a-Si_x_C_y_) and SOI substrates. The results presented show a gauge factor (GF) of 48 for the a-Si_x_C_y_ sensor and of 22 for the p-type SOI sensor. For thermal stability, the a-Si_x_C_y_ sensor achieved a change of 35 ppm/K, where the p-type achieved a TCR (temperature coefficient of resistance) of 140 ppm/K. The tests were made at a temperature of up to 250 °C.

### 1.4. Capacitive Sensors

Capacitive sensors, compared to piezoresistive sensors, have lower temperature drift because the capacitance is usually not fabricated with a diffusion process. Conventional capacitive sensors are usually manufactured as a standard parallel-plate capacitor [22]. A change at the pressure-sensitive membrane, located on one or both sides of the sensor, results in a change of the electrode gap or the dielectric property of the medium between the plates (dielectric constant: ε_r_), and this leads to a change of the capacitance. Capacitive pressure sensors are suitable for a wide pressure range, from 250 Pa up to high pressures of up to 70 MPa [7]. In addition, they feature high pressure sensitivity, high accuracy, low power consumption, and high shock resistance [28,29]. However, their parasitic and stray capacitances make it difficult to measure the capacitance and linearize the output signal [23]. Moreover, the fabrication of capacitive sensors usually requires critical pretreatments, complex equipment, and high process temperatures required for packaging methods such as anodic bonding, silicon–silicon direct bonding (fusion), and glass frit bonding [30,31]. In addition, such bonding methods complicate the electrical connection of the sensor electrodes to the contact pads, which has become a major challenge for the design and fabrication of these sensors [30]. An example of a capacitive pressure sensor with a membrane made of SiC on a silicon substrate is presented in [28]. An operating temperature of 400 °C was achieved with a nonlinearity error of 2.1%, a hysteresis of 3.7%, and a strong temperature dependence of the output signal. Similar structures and properties are shown in [32,33]. These also suffer from a particularly large shift of the base capacitance from about 45 pF at room temperature to about 20 pF at 500 °C, probably due to the different thermal expansion coefficients of the different materials (silicon substrate and SiC membrane). Measurements of a capacitive pressure sensor made of SiC up to 574 °C with a sensitivity and nonlinear error of 7.2 fF/psi and 2.4% are shown in [34]. Compared to [28], the same material was used for the substrate and membrane, resulting in a significant improvement in thermal stability (0.05%/K). A high operating temperature of up to 600 °C (850 °C for a short time) is demonstrated in [35]. Here, too, the same material was used for the substrate and membrane. The sensor consists of three sintered aluminum oxide ceramic plates with silver metallization. The evaluation of the capacitance change is measured with a resonance approach. The sensor capacitance forms an LC resonant circuit with a coil, the frequency of which is pressure dependent. However, the measured temperature dependence of the resonant frequency in the selected measuring range (25 °C to 600 °C, 1 to 5 bar) is significantly higher than the pressure sensitivity itself. In addition, a measurement error of more than 10% FS is specified. Table 1 shows the main advantages and disadvantages of the most common pressure and force sensor technologies.

The aim of this work was to develop a low-cost, miniaturized pressure, and force sensor that exhibits very good thermal stability without additional mechanical or electrical compensation. Since piezoelectric sensors are only suitable for dynamic measurements, piezoresistive sensors are not suitable for high temperature ranges without additional high manufacturing costs due to temperature-dependent semiconductor effects, and since optical sensors have a high thermal sensitivity, we decided to develop capacitive sensors. This paper presents a new fabrication technology for capacitive force and pressure sensors. The fabricated sensors are shielded by an integrated Faraday cage on the chip itself. Moreover, they are characterized by their high sensitivity, as the capacitance change is over 100% compared to the base capacitance. By using gold–silicon (Au-Si) or alternatively aluminum–silicon (Al-Si) eutectic bonding, operating temperatures of up to 350 °C for Au-Si and up to 500 °C for Al-Si are achieved. The measurements show an excellent linearity of the inverse capacitance value of 99.992% FS at 350 °C and 99.965% FS at 500 °C. A very good thermal stability is observed, with a drift of only 0.006%/K FS at 350 °C and of −0.0027%/K FS at 500 °C without additional compensation. The full scale (FS) is defined as the force corresponding to a 100% change in capacitance. The greatest benefit of the manufacturing process over conventional methods is that the process parameters are independent of the sensor design. This means that different sensor designs for different ranges can be produced in the same batch.

## 2. Materials and Methods

### 2.1. Sensor Design

Based on the findings of the previous section, our observations can be summarized as follows:

- A majority of the presented capacitive sensors work with extremely thin membranes on small areas. This leads to low basic capacitances and excludes the use as a force sensor;
- Only capacitive sensors which consist completely of one material show a sufficiently low temperature dependence;
- Pure SiC sensors with a membrane and base body made of SiC show good performance data but are relatively expensive and complex due to the related material and process costs;
- A low-noise readout of the sensors is generally a challenge, as the capacitance changes and base values may be small.

**Figure 1:**
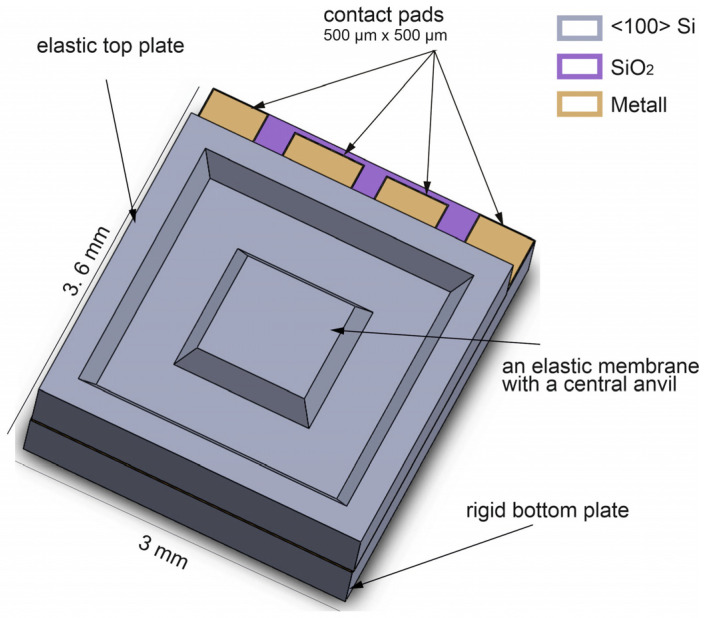
Three-dimensional sketch of an all-silicon capacitive pressure and force sensor.

**Figure 2:**
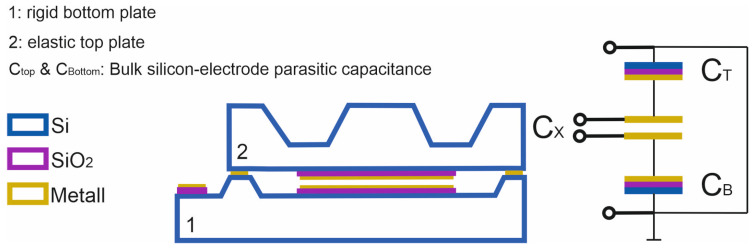
Cross-sectional side view (**left**) and electrical equivalent circuit (**right**) of the sensor.

Taking these points into account, the following concept was developed (Figure 1 and Figure 2). The sensor is designed as a standard plate capacitor consisting of two different silicon chips, one carrying an elastically deformable top electrode, and the other one a rigid bottom electrode. Both chips are mounted via Au-Si or Al-Si eutectic bonding. Since monocrystalline silicon is known for its very good thermal, mechanical, and elastic properties, silicon is the preferred starting material. In addition to its high hardness, the monocrystalline material exhibits virtually no plastic deformation to fracture, and this is very advantageous for the fabrication of spring elements [36]. By using silicon as a purely mechanical component and not as a semiconductor, temperature-dependent semiconductor effects are avoided. In order to minimize the thermal stresses caused by the different temperature coefficients of the different materials, both sensor plates are made of the same material (silicon). Therefore, the sensor structure has a quasi-monolithic design, which increases the mechanical and thermal stability.

The chip components are bonded together by eutectic bonding. Compared to other bonding methods, such as silicon–silicon direct bonding and anodic bonding, eutectic bonding does not require complex equipment, high voltage, and critical pretreatments. In addition, eutectic bonding has a very high mechanical strength. For the Au-Si eutectic, the tensile strength is around 255 MPa, and for Al-Si, it is around 170 MPa [37,38]. In addition, higher operating temperatures up to 500 °C are possible due to the eutectic bonding process. Finally, eutectic bonding welds both sensor components together, and, thus, they are mechanically and electrically connected. As a result, the capacitive measuring cell is surrounded by silicon material, which forms a Faraday cage around the measuring electrodes. The sensor is thus shielded from external electrostatic interference. An important aspect of this design is that the silicon material forms a third electrode that forms parasitic capacitances with the two measurement electrodes. This electrode can be brought to a defined potential, with the advantage that the measurement electrodes can be tapped directly (floating mode) and that the influence of the parasitic capacitances on the measurement signal can be avoided. Thus, the output signal has a much higher signal swing compared to conventional capacitive sensors. Figure 2 provides a sketch of the equivalent circuit of the sensor.

To increase sensitivity, the electrode spacing in rest is designed to be 5 µm. Since the capacitance is inversely proportional to the electrode distance, a capacitance change of 100% can be expected by dislocating the top electrode to half the initial distance. Due to the small electrode spacing, a quasi-displacement-free measurement is guaranteed.

### 2.2. Fabrication

**Figure 3:**
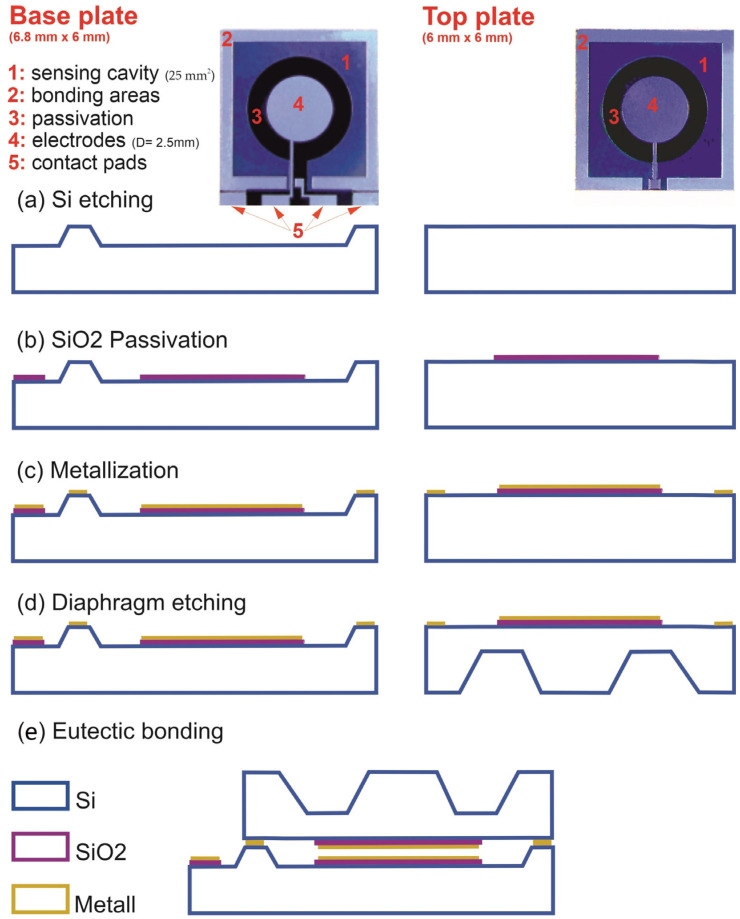
Top view of the front sides of the base and top plate with the schematic cross-sectional view of the clean-room process flow.

The fabrication of the sensors is based on standard and industrial MEMS technology using monocrystalline <100> silicon. Figure 3 shows a simplified sketch of the clean-room fabrication process. To fabricate the force/pressure sensor, two silicon wafers are processed simultaneously for the base plate and the top plate. First, both wafers are etched with KOH to pattern the sensing cavity (Figure 3a). The KOH step is followed by a thermal oxidation and one lithography step to structure the insulating SiO_2_ layer (Figure 3b). For eutectic bonding, the silicon dioxide (SiO_2_) is removed from the outer edge of the chip (bonding area). This is followed by a metallization step and lithography (Figure 3c) to create the electrodes on the SiO_2_ layer. In the same step, the eutectic metal (gold or aluminum) is also deposited and patterned on the elevated bonding areas of both chips to later create a eutectic bond with the top plate. In process steps (a) to (c), the top plate is patterned in the same way as the base plate. Now the top plate is etched from the backside with KOH to form an elastic membrane with a central anvil on the backside of the chip (Figure 3d). The central anvil improves the force coupling and, thus, the linearity of the signal, while reducing temperature drift. Finally, the top plate is flipped over, aligned with the base plate, and eutectically bonded. The top electrode is guided via a conductive path to a contact pad, which is located in the suitably provided area of the base plate via an appropriately sized recess that is electrically connected to the contact pad of the base plate by bonding.

#### 2.2.1. Gold–Silicon Eutectically Bonded Sensors

**Figure 4:**
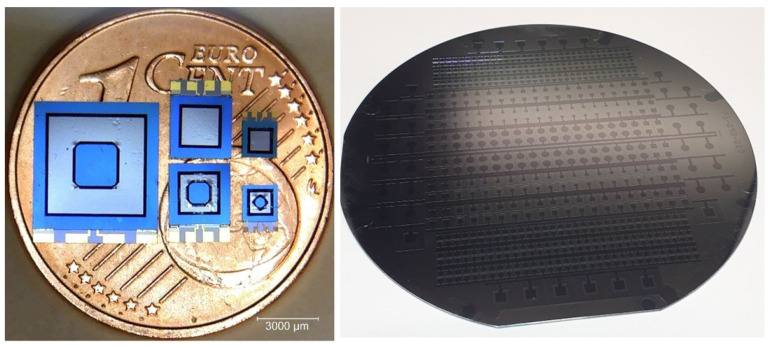
Top view of different sensors manufactured on the same wafer in Run 1. (**Left**): Type F8 (chip size 6.8 mm × 6 mm). Type F2 with and without anvil (chip size 3.8 mm × 3 mm). Type F7 with and without anvil (chip size 2.25 mm × 1.7 mm) “Reprinted with permission from Ref. [39]. 2021, IEEE”. (**Right**) Base wafer.

Two different runs are produced with this manufacturing process. In the first run, Au-Si eutectic bonding is used for operating temperatures up to 300 °C. To illustrate the flexibility of the fabrication process, three different sensor dimensions with different sensor designs are fabricated on the same wafer (Figure 4). The sensor designs differ with the shape of the electrode (circular or square), with the membrane structure (with or without anvil), and also with the bonding area. Besides the membrane thickness, the measuring range can also be adjusted by the bonding surface (membrane area). The F10-type, for example, has the same chip dimensions (6.8 mm × 6 mm) and the same diaphragm thickness as the F8 type, but it has more than three times the bond area, so the maximum force to be measured is up to six times higher than for the F8 type. Table 2 shows the notable differences of the fabricated sensors.

**Figure 5:**
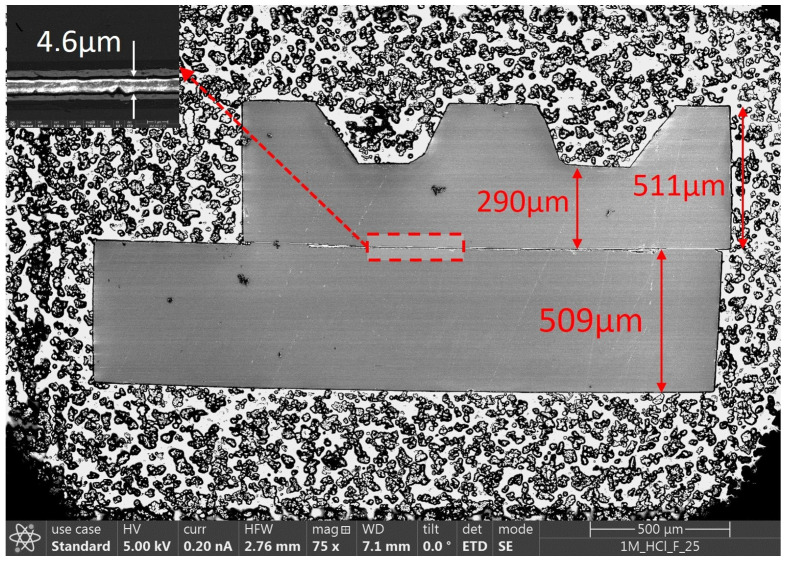
Microscope cross-sectional image of an F7 force sensor “Reprinted and adapted with permission from Ref. [39]. 2021, IEEE”.

For Au-Si eutectic bonding, the two chips are brought into contact and heated to 400 °C, with a contact force of 190 N, over a period of 300 s. The resulting distance between the capacitive electrodes is about 5 µm, resulting in a base capacitance between 1.2 pF and 6.6 pF (depending on the sensor type). This small distance between the electrodes increases the sensitivity of the sensor and ensures an almost displacement-free force measurement. Figure 5 shows, as an example, the cross-sectional image of an F7 sensor.

#### 2.2.2. Redesign with Aluminum–Silicon and Gold–Silicon Eutectically Bonded Sensors

In the second pass, partly to simplify the backend, new masks were designed with only one sensor dimension. Since temperature changes are expected to have a greater effect on the performance of sensors with larger membrane areas, we chose an F8 sensor, which has the largest membrane area, i.e., 25 mm^2^. The sensor was redesigned with larger electrodes than in Run 1 to achieve a larger change in capacitance. As explained earlier, different sensor dimensions were fabricated on the wafer in the first run. To avoid possible instabilities and predetermined breaking points in Si wafers during the process, membrane patterning was performed in a KOH etch box in the last process step. In contrast to the first run, the membranes in the second run were already etched in KOH etch tanks in the first process step, together with the structuring of the measurement cavity (3. a). This saves one process step (3. d) and significantly increases the productivity of the process. Two pairs of wafers, one for Au-Si and one for Al-Si eutectic bonding, were fabricated. The bonding parameters are the same as in the first pass. For Al-Si eutectic bonding, the bonding temperature is 650 °C instead of 400 °C.

Different temperatures were tested for the eutectic Al-Si compound. The eutectic temperature of 577 °C given in the Al-Si phase diagram [40] is not sufficient for bonding the sensor. The best results were obtained at 650 °C and above. To protect the Al electrodes and Al contact pads from oxidation at higher temperatures, they are coated with a platinum (Pt) layer. However, after bonding, we found that Pt forms a phase with Al. Murarka et al. reported in [41] that platinum also forms various phases with aluminum at lower temperatures, such as 250 °C. Due to the Al-Pt phase formed after eutectic bonding, neither wire bonding nor electric welding of wires is possible anymore. For this reason, press contacts are used for characterization.

## 3. Results

**Figure 6:**
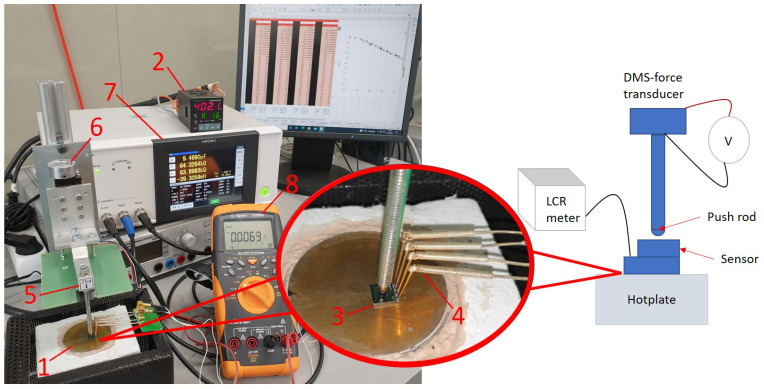
Test setup with (1) hotplate, (2) temperature controller, (3) sensor, (4) press contacts, (5) force transducer with a stainless-steel push rod, (6) force adjuster, (7) LCR meter, and (8) voltmeter.

For the initial characterization of the F8 sensors from the first and second run, a high-temperature test setup is required. For this purpose, a molybdenum heating plate coated with gold was specially manufactured. This hotplate can be heated up to 900 °C. The temperature is controlled by an INKBIRD temperature controller (ITC-100VL) and precisely measured by a Pt-1000 temperature sensor and a type K thermocouple integrated into the hotplate. As shown in Figure 6, the sensor is mounted on the hotplate, and force is applied via a stainless-steel push rod. The force is measured using a conventional 20 N strain gauge force transducer, with a sensitivity of 0.002 N/mV, mounted on the cold side of the rod. Table 3 shows a comparison of the data between the measured sensors.

**Figure 7:**
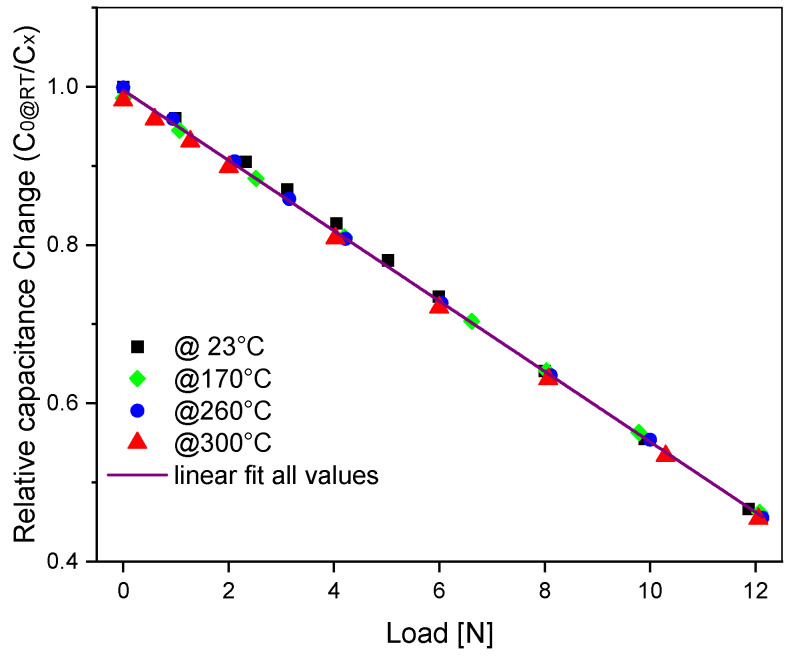
Capacitance change of an F8-type sensor (Run 1) vs. load at 23 °C, 170 °C, 260 °C, and 300 °C.

Figure 7 shows the measurements of an F8-type Run 1 sensor with a 300 µm thick membrane at room temperature, 170 °C, 260 °C, and 300 °C. All values are normalized with the same value of the room-temperature zero-load capacitance, C_0_, so no temperature compensation was applied. The capacitance change was measured with an LCR meter (Rohde & Schwarz HM8118) at a frequency of 10 kHz, a voltage amplitude of 1 V, and a resolution of 0.01 pF. The sensor is connected to the LCR meter by four shielded RF coaxial cables connected to the sensors by thermal welding. The substrate electrode is connected to the cable shield. The capacitance is 5.46 pF at zero load and 11.84 pF at 12.06 N. A nonlinearity error of the inverse capacitance value of 0.045% FS at room temperature and 0.005% FS at 300 °C is measured. Temperature drifts of only −0.0062% FS/K and −0.006% FS/K are achieved without load and at full load (FS), respectively.

The sensors from the second run are connected via four silver-coated stainless-steel press contacts, which are connected to the LCR meter via shielded RF coaxial cables. The substrate electrode is connected to the cables’ shield. The sensor capacitance is measured with an LCR meter (HIOKI IM3536) operating at a frequency of 10 kHz and 30 kHz, a voltage amplitude of 1.5 V, and a resolution of 0.01 pF.

**Figure 8:**
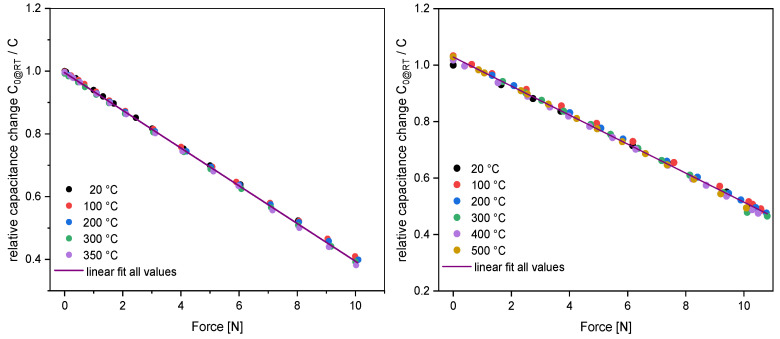
Comparison of the characterization of Au-Si and Al-Si sensors from Run 2. (**Left**), capacitance change of an Au-Si F8-type sensor vs. load up to 350 °C. (**Right**), capacitance change of an Al-Si F8-type sensor vs. load up to 500 °C.

Figure 8 shows the high temperature characterization of the Au-Si and Al-Si F8-type sensors from the second run. For the Au-Si sensor, the zero-point capacitance at RT is 9.55 pF and 19 pF at about 8 N (FS). The characteristics of the inverse of capacitance versus force are recorded at 20 °C, 100 °C, 200 °C, 300 °C, and 350 °C. The measurements extend beyond the defined FS range (8 N), up to a force of 10 N, which corresponds to a 120% change in capacitance. Nevertheless, the results show a high linearity of the sensor characteristics of 99.987% at RT, 99.996% at 300°, and 99.992% at 350° and a very low thermal drift of −0.001%/K at 350 °C without load and 0.006% FS/K at 350 °C and full load (10 N).

The capacitance of the Al-Si F8-type at 23 °C is 9.53 pF at zero load and 19.11 pF at 10,23 N (FS). Measurements were made at 23 °C, 100 °C, and with 100 °C steps up to 500 °C. The results show a nonlinearity error of 0.14 %FS at 23 °C and 0.035 %FS at 500 °C. Temperature drift values of only 0.0061 %/K without load and −0.0027 %FS/K with FS load at 500 °C are achieved. Both graphs show different slopes, because both sensors have different membrane thicknesses of 200 µm and 240 µm, respectively (see Table 3).

**Figure 9:**
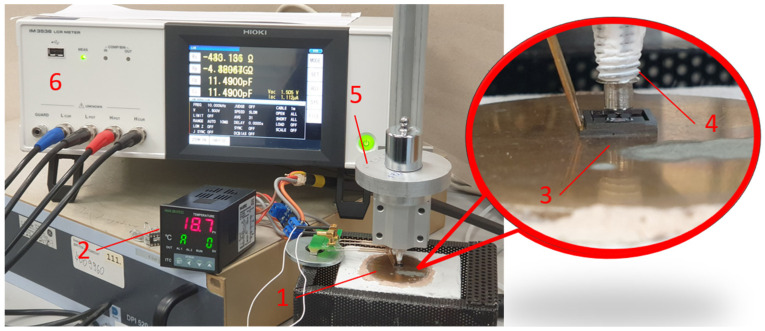
Repeatability and hysteresis test setup with (1) hotplate, (2) temperature controller, (3) sensor, (4) stainless-steel push rod with an 800 µm stainless-steel ball, (5) weight loader unit with a 100 g weight, and (6) LCR meter.

Figure 9 shows the test setup for measuring repeatability and hysteresis. A different loading method by adding and removing weights was chosen because it ensures repeatable loading, as long as the friction is properly minimized. A weight loader unit connected to a stainless-steel push rod is inserted via a linear ball bearing and positioned on the sensor. The force is transmitted to the sensor via an 800 µm diameter stainless-steel ball located at the end of the push rod. The initial weight of the test setup is 172.1 g due to the load unit.

**Figure 10:**
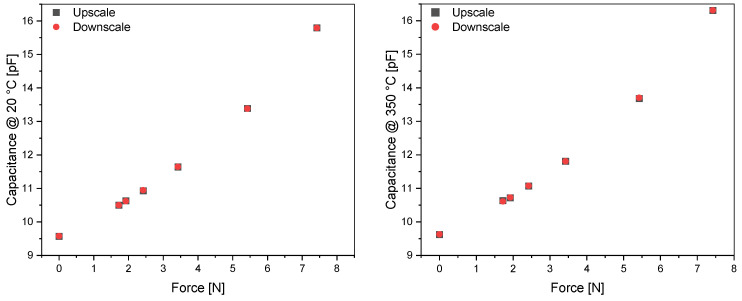
Hysteresis measurement of an Au-Si F8-type sensor (Run 2): (**Left**) at 20 °C and (**right**) at 350 °C.

Figure 10 shows the hysteresis measurement of a type F8 sensor fabricated in the Au-Si Run 2. Measurements are taken at RT and 350 °C, with seven points defined by the weights (0, 172.1 g, 192.1 g, 242.1 g, 342.1 g, 542.1 g, and 742.1 g) up and down the scale.

The hysteresis is determined by the maximum absolute difference between upscaling and downscaling, as follows:

(1) $$u_{hysteresis}=Max\left|x_{upscale}-x_{downscale}\right|$$

**Figure 11:**
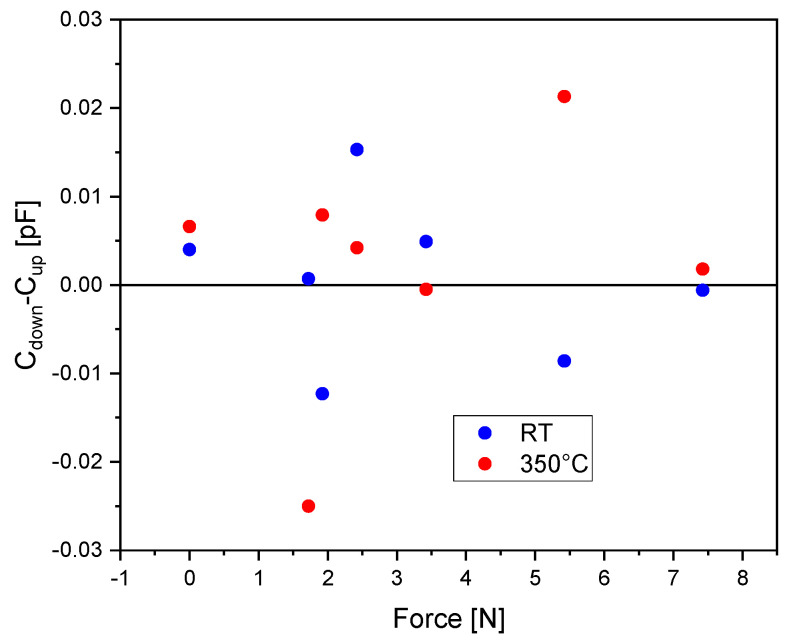
The deviation between the measured capacitance values (C_down_-C_up_) vs. force.

The measured hysteresis is 0.015 pF at room temperature and 0.025 pF at 350 °C. Nevertheless, these values cannot be attributed to a hysteretic process, as shown in Figure 11. The deviation between the measured capacitance values, C_down_-C_up_, shows a random behavior and is probably due to a noise effect and not due to hysteresis. Therefore, we conclude that the real hysteresis, if existent, cannot be measured because it is lower than the measurement accuracy of our setup.

**Figure 12:**
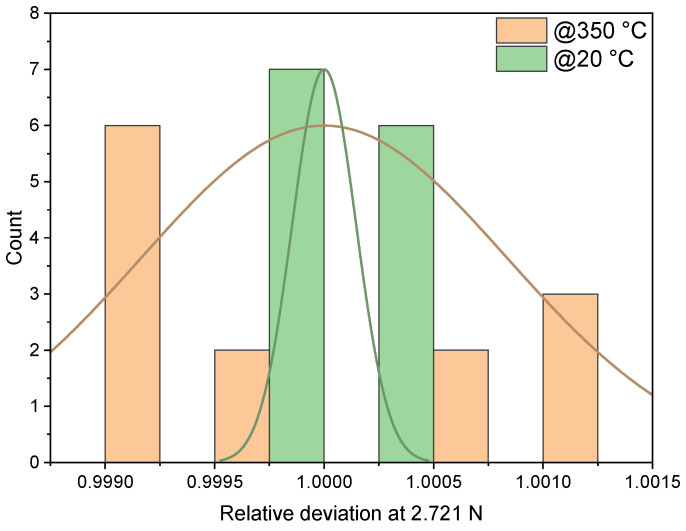
The relative standard deviation of an Au-Si F8-type sensor (Run 2) at room temperature and at 350 °C and a force of 2.721 N.

The same test setup is used for repeatability measurements. To minimize possible errors due to friction and the repositioning of the weight loader unit, the loader unit is not moved once it is attached to the sensor. This sets the loader unit weight of 172.1 g as the zero point for subsequent measurements. A 100 g weight (272.1 g total) is used to check the non-repeatability of the sensor. The measurements are repeated thirteen times at room temperature and at 350 °C, as shown in Figure 12. The relative standard deviation (RSD) is 0.0146% at room temperature and 0.0837% at 350 °C.

## 4. Discussion

The fabrication process presented here enables us to manufacture force and pressure sensors, with the complete structural material made of silicon. With the sensor design presented, hardly any thermal stresses occur. Temperature-dependent semiconductor effects also do not occur in the sensors since silicon is used only as a mechanical material and not as a semiconductor. Temperature changes only affect the coefficient of thermal expansion and Young’s modulus and are low in effect for silicon. Therefore, in most cases, no temperature compensation is required. This manifests itself especially in the stability of the zero-point capacitance and the force sensitivity over temperature, as shown by the measurements. Due to the flexibility of the eutectic bonding step in the fabrication process, an operating temperature of up to 600 °C is conceivable, limited, in general, only by the plastic deformation of silicon [42] at a high temperature. The fabricated capacitive sensor electrodes are shielded by a Faraday cage constituted by the surrounding silicon material. In addition, the sensors have the following properties: an expected good chemical resistance and a proven high reproducibility, high sensitivity, and high mechanical strength.

It must still be mentioned that the nonlinearity and reproducibility of the used force transducer for the low end of measurements at 0.02% FS and the accuracy of the LCR meter influence the measurement accuracy. Other possible sources of error, such as the cooling of the force sensor by the stainless-steel rod, as well as the heating of the strain gauge force transducer and the resulting thermal error, have not yet been investigated. Nevertheless, the measurement results are constant even over longer measurement periods (up to hours) and provide promising results. The sensors presented here have a high linearity of over 99.99% FS and excellent thermal stability of less than 30 ppm/K FS at 500 °C. The sensors thus have 10 to 100 times better thermal stability than the other published works, as shown in Table 4. In our own previous work [39], comparative measurements of different sensor types from the first run with and without membrane anvil and at frequencies between 1 kHz and 100 kHz were presented. All presented measurements show no influence of the operating frequency of the LCR-meter on the linearity and temperature sensitivity of the sensors. The temperature sensitivity for the sensor without an anvil is higher by a factor of 10 than for sensors with a membrane anvil.

Furthermore, the high range of capacitance change, which is more than 100% of the no-load capacitance, guarantees the high sensitivity of the sensor. The flexibility of the manufacturing process allows for an adjustment to the sensor design and the required thickness of the membrane, which, in turn, allows for the fabrication of different sensors for different measurement ranges (from mN to kN) even in the same batch.

A further test setup is planned for the future to minimize thermal errors and to characterize the sensors at higher forces. In addition, a new approach to patterning through silicon vias (TSVs) will be developed and integrated into the existing process. This will enable bonding at the wafer level and the ability to produce absolute sensors. Furthermore, an appropriate metal stack has to be found that enables eutectic aluminum–silicon bonding at 650 °C without affecting the wire bonding capability.

## 5. Conclusions

In this manuscript, an innovative manufacturing process for the production of capacitive pressure and force sensors with excellent thermal stability for high-temperature applications was presented. Compared to conventional methods, the greatest benefit of this manufacturing process is that different sensor dimensions can be produced in the same batch for a wide measuring range, from mN to kN. The sensors are completely fabricated out of silicon, which is particularly suitable due to its highly reproducible elasticity properties. This is particularly evident from the almost hysteresis-free measurements. Measurements show high linearity of over 99.99% FS and excellent thermal stability of less than 30 ppm/K FS at 500 °C. In addition, the high range of capacitance change, which is greater than 100% of the no-load capacitance, ensures the high sensitivity of the sensor. The repeatability for the F8 sensor was determined to be 0.015% (RSD) at room temperature and 0.084% (RSD) at 350 °C. A hysteresis could not be measured within this accuracy. Deviations from the ideal temperature-independent behavior are hardly measurable with the measurement technology used in the study.

## Figures and Tables

**Table 1:** The main advantages and disadvantages of the most common pressure and force sensor technologies.

Sensor Principle	Optical	Piezoelectric	Piezoresistive	Capacitive
Advantages	High accuracy High resolution Insensitive to electromagnetic interference High operating temperature	Ease of integration High operating temperature Wide measuring range High resolution	Low cost Simple signal processing circuit miniaturized Good linearity	High sensitivity High accuracy Low power consumption High shock resistance
Disadvantages	Temperature sensitive Complex signal processing circuit	Complex electronic interface Only dynamic measurement expensive	High power consumption Sensitive to temperature and mechanical stress	Susceptible to parasitic and stray capacitance Complex fabrication Complex signal processing circuit

**Table 2:** Data from different sensors with a central anvil manufactured on the same wafer in the first run.

Chip Type	F2	F3	F6	F7	F8	F10
Chip size (mm^2^)	3.8 × 3	3.8 × 3	2.25 × 1.7	2.25 × 1.7	6.8 × 6	6.8 × 6
Diaphragm thickness (µm)	300	300	300	300	300	300
Capacitance C_0_ (no load) (pF)	4.5	1.2	1.6	1.2	5.4	6.6
Max force (N)	80	80	200 *	200 *	12	63

* Computed value.

**Table 3:** Data of the characterized F8-type sensors.

Chip Type	F8 Run 1	F8 Run 2	F8 Run 2
Bonding material	Au-Si	Al-Si	Au-Si
Chip size (mm^2^)	6.8 × 6	6.8 × 6	6.8 × 6
Diaphragm thickness (µm)	300	240	200
Capacitance C_0_ approx. (pF)	5.46	9.53	9.55
Max. force (at 100% capacitance change) (N)	11	10	8

**Table 4:** Performance comparison with other publications.

	[8]	[20]	[26]	[27]	[28]	[34]	F8-Type Au-Si	F8-Type Al-Si
Sensor principle	optical	piezoelectric	piezoresistive	piezoresistive	capacitive	capacitive	capacitive	capacitive
Thermal stability	15.7 pm/°C	≤5%	0.082% FS	0.0035%/K	high	0.05%/K	−0.001%/K	0.006%/K
Max Temperature	1000 °C	650 °C	200 °C	250 °C	400 °C	574 °C	350 °C	500 °C
Nonlinearity	-	≤1%	±0.14% FS	-	2.1%	2.4%	0.008% FS	0.035% FS

## Data Availability

Publicly available datasets were analyzed in this study. This data can be found at “https://bwsyncandshare.kit.edu/s/w6bdzKi8kcLRQnR (accessed on 8 April 2023)”.
